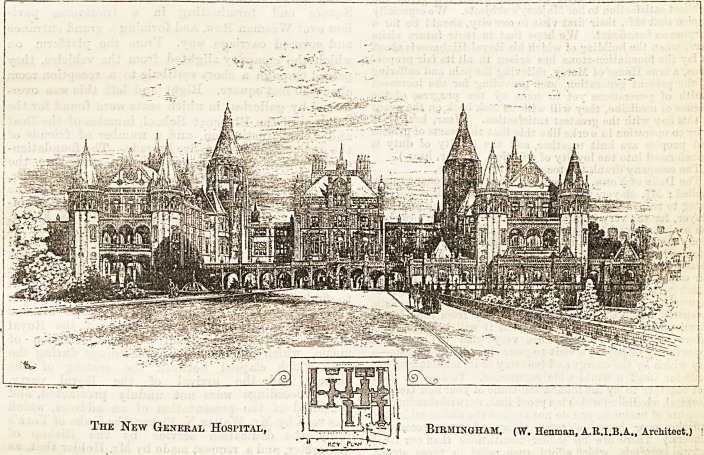# The Royal Visit to Birmingham

**Published:** 1894-09-15

**Authors:** 


### The Institutional Workshop.

### THE ROYAL VISIT TO BIRMINGHAM.

The proceedings at Birmingham on the 8th instant, when the Duke and Duchess of York laid the foundation-stone of the New General Hospital there, were worthy of the best traditions of the Metropolis of the Midlands. The weather had been unsettled during the previous days, and much anxiety was evinced lest rain should mar the proceedings. Fortunately, the day was beautiful, and the miles of streets through which the royal procession passed were crowded with people. Birmingham was entered by the royal party at Saltley, one of the poorest portions of the city. Here there are a great number of small two-storey houses, and it was touching to notice the attempts at decoration made by each of the householders, however humble the dwelling. The people, who had come in great crowds from the surrounding districts, were present in thousands, and they waited patiently for hours, in order to get a glimpse of the Duke and Duchess of York. Everywhere enthusiasm prevailed, and there can be no doubt of the sincerity of the appreciation which the Duke of York expressed on behalf of the Duchess and himself, of the splendid and enthusiastic reception which they had throughout the day's proceedings. It would be out of place for us, on the present occasion, to endeavour to give a lengthened report of the many incidents which happened on a long and busy day. Suffice it to say that everything went off without a hitch, and that from the reception in the Town Hall to the laying of the foundation-stone in the afternoon the imposing ceremonial passed off with the most perfect *éclat*. This happy result is in a great measure due to the exceptional ability of the Mayor, Mr. G. J. Johnson, who was most ably seconded by the Deputy Mayor, Mr. Alderman Lawley Parker, Mr. Alderman Clayton, and the other members of the Reception Committee. The Mayor surpassed himself—and that is saying much—at the luncheon, where his well-chosen words and eloquent speech, combined with the warm response of the Duke of York, met with the heartfelt approval of all present.

The New General Hospital, Birmingham. (W. Henman, A.R.I.B.A., Architect.)

### The Speeches at the Luncheon.

The Mayor rose, amid cheers, to propose the toast of "The Queen." His Worship said: I have the honour, your Royal Highnesses, my Lords, Ladies, and Gentlemen, of proposing the first toast on the list, the health of her Majesty the Queen. For fifty-seven years this toast has been proposed at every assembly such as this. Fifty-seven years ago it was proposed with hope and sympathy for a lady called to a destiny so august as to preside over this empire. Every year since it has been honoured with increasing admiration of the manner in which her Majesty has discharged her multifarious duties. As a constitutional Sovereign she has displayed a wisdom and a discretion which have won the respect of every political party in this country. Her domestic life has been an example to her subjects, and her quick womanly sympathy in every calamity that has befallen her subjects, public and private, has elicited our admiration and affection. As the Poet Laureate has said,

> "A thousand claims to reverence close
> In her, as mother, wife, and Queen."

But this afternoon we drink the toast with an added pleasure. You, sir, and you, your Royal Highness (the Duchess), stand on the steps of the Throne, and we, in many and varying ranks and degrees, are her Majesty's humbler subjects. But to you and to us her Majesty is our Queen, and you will join us in honouring the toast I now propose—"Her Majesty the Queen."

The toast was drunk with enthusiasm, the band playing the National Anthem.

The Mayor: My Lords, Ladies, and Gentlemen,—I have now to propose "The Health of the Prince and Princess of Wales, the Duke and Duchess of York, and the rest of the Royal Family" The Prince and Princess of Wales are no strangers in the city of Birmingham. (Hear, hear.) We have had the pleasure of welcoming the Prince three times and the Princess twice, and with them, as with the rest of the Royal Family, we know their readiness to participate in such functions as that we celebrate to-day, and the nearty welcome with which they are received among all classes of the people. We regret that the health of her Royal Highness the Princess of Wales has not permitted her to appear amongst us so frequently as we could have hoped; but we all recollect the graciousness and dignity with which the Prince and Princess of Wales have discharged every function which they have had to perform. We have here to-day the Duke and Duchess of York. (Loud cheers.) We all rejoice to see her Royal Highness the Duchess of York in such excellent health after an event which has given the liveliest satisfaction to her Majesty's subjects. We especially rejoice that this, their first visit to our city, should be for a purpose so beneficent. We hope that in their future visits here, when the building of which his Royal Highness is about to lay the foundation-stone has arisen in all its fair proportions, a true House of Mercy, relieving the pain and suffering of the present generation, and providing for the increased health of generations yet to come by the progress of the science of medicine, they will with us look back on the event of this day with the greatest satisfaction. (Hear, hear.) It is by co-operation in works like this that the hearts of princes and peoples are knit together, and the loyalty of duty is transformed into the loyalty of affection. (Hear, hear.)

The company drank the toast with three cheers.

The Duke of York, who spoke in firm and resonant tones, replied: Mr. Mayor, my Lords, Ladies, and Gentlemen,—In rising to respond to this toast, let me first thank you, Mr. Mayor, for the very kind way in which you have proposed the health of my father and mother, and the other members of my family, and I thank you most heartily for the cordial manner in which you have received this toast. I have much pleasure in expressing on behalf of the Duchess and myself our warmest thanks for the words—the most kind words—which you have used towards us. I cannot find words to express our deep appreciation of the splended and enthusiastic reception which we have received to-day from the city of Birmingham. I can assure you that it will be a long time before we forget it. We are very glad to have this opportunity of paying a visit to your city of Birmingham—a city which by the energy and industry of its inhabitants, has made for itself a world-wide reputation. To-day we have met together to lay the foundation-stone of your new General Hospital, a building which is a proof that, notwithstanding the pressure of business, you do not neglect the claims which the suffering poor have on your time and your resources. (Cheers.) There are no nobler institutions than our large general hospitals, which afford such relief to those struck down by sickness or injury, and the Duchess and I rejoice that our first visit to Birmingham should connect us with this excellent work. (Hear, hear.) It is not possible for me on such an occasion as this to allude to the many industries that are carried on in Birmingham, but I believe I am right in saying that your manufactures are so various and so useful that they supply wants that exist in every country and in every home. Before I sit down I should like to propose a toast, to which I am sure you will all heartily respond, and that is "Success to the City of Birmingham, and Prosperity to its Industries." May it always maintain its high position for the excellence of its manufactures. I will ask you to drink with this toast the health of the Mayor, Mr. Johnson—(cheers)—and I am sure I express the feelings of all present when I say that we thank him most warmly for his kind hospitality to us on this occasion. (Cheers.)

The toast was received with enthusiasm.

The Mayor: Your Royal Highnesses, my Lords, Ladies, and Gentlemen,—The gracious manner in which this toast has been proposed, and the cordiality with which it has been received quite incapacitate me from making any adequate response. It is one of the occasions on which I regret that I am not connected with the industries of Birmingham that I might, on behalf of the city, say with greater knowledge something proper to the occasion. I am quite sure that we all recognise the heartiness of the wish which your Royal Highness has expressed for the prosperity of the city. (Hear, hear.) For myself, I can only say in the words which Shakspeare puts into the mouth of Hamlet, "I am poor in thanks, but I thank you." (Cheers.)

### The Stone-laying.

The ceremony of laying the foundation-stone of the New General Hospital took place in the largest tent we have ever seen. It was magnificently proportioned, gorgeously decorated, and crowded with an audience of upwards of 3,000 people. The pavilion was provided by the munificence of Mr. J. C. Holder, chairman of the New Hospital, and extended the whole length of the hospital site, forming a structure about 300 feet long by 85 feet wide, the canvas roof being supported by a line of eight masts, rising to a height of 40 feet. The seating was arranged along three sides of the structure, the St. Mary's Square end terminating in a transverse pavilion over Weaman Row, and forming a grand entrance and covered carriage way. From the platform on which the company alighted from the vehicles, they passed through a short vestibule to a reception room about 40 feet square. Right and left this was overlooked by galleries, in which seats were found for the children of the Bluecoat School, inmates of the Deaf and Dumb Institution, and a number of friends of the police officers and fire brigade. The foundation-stone was of polished red granite, and bore the following inscription: "On the 8th September, A.D. 1894, this stone was laid by H.R.H. the Duke of York, K.G. William Henman, Architect; John C. Holder, Chairman; Walter N. Fisher, Honorary Secretary." Despite the thousands of people present, owing to the admirable organisation of the large number of stewards under the direction of Mr. S. D. Balden, and Mr. Howard Collins, House Governor of the General Hospital, who, we were glad to find, has won golden opinions in Birmingham, every person present easily found his place without confusion or discomfort. The band of the Royal Marine Artillery played an excellent selection of music, which enlivened the proceedings during the time which elapsed between the seating of the visitors and the arrival of the Royal party. The proceedings were not unduly protracted, and consisted of the presentation of an address, which was read by Mr. Walter N. Fisher, the Duke of York's reply, the dedication service by the Bishop of Worcester, and a request made by Mr. Holder that, as a lasting memento of the visit, one of the wards might be named after Her Royal Highness the Duchess of York, and that it might be called the "Princess May" Ward. This request was graciously acceded to, and the Duke of York then laid the stone with evident enjoyment. When the stone had been properly adjusted His Royal Highness said, "In the name of the Father, the Son, and the Holy Ghost, I declare this stone to be well and truly laid." A fanfare of trumpets from the band, followed by general cheering, concluded the ceremony, and as the Royal party proceeded down the pavilion the band played "God Bless the Prince of Wales."

We shall have much to say on a future occasion of the plans of the new buildings, and have nothing to add now except to point out, as will be seen from the view which we publish to-day, that the elevation of the new hospital is one of the most beautifully designed buildings ever dedicated to the purposes of a hospital. Great praise is due to the committee and officers who had charge of the arrangements, and we congratulate them upon a triumphant success.

## Figures and Tables

**Figure f1:**